# The Barbados Emergency Ambulance Service: High Frequency of Nontransported Calls

**DOI:** 10.1155/2012/659392

**Published:** 2012-11-07

**Authors:** Sherwin E. Phillips, Pamela S. Gaskin, David Byer, W. L. Cadogan, Andrew Brathwaite, Anders L. Nielsen

**Affiliations:** ^1^Emergency Ambulance Service, Queen Elizabeth Hospital, Barbados; ^2^Faculty of Medical Sciences, The University of The West Indies Cave Hill Campus, St. Michael, Barbados

## Abstract

*Objectives*. There are no published studies on the Barbados Emergency Ambulance Service and no assessment of the calls that end in nontransported individuals. We describe reasons for the nontransport of potential clients. *Methods*. We used the Emergency Medical Dispatch (Medical Priority Dispatch System) instrument, augmented with five local call types, to collect information on types of calls. The calls were categorised under 7 headings. Correlations between call types and response time were calculated. *Results*. Most calls were from the category medical (54%). Nineteen (19%) percent of calls were in the non-transported category. Calls from call type Cancelled accounted for most of these and this was related to response time, while Refused service was inversely related (*P* = 0.01). *Conclusions*. The Barbados Ambulance Service is mostly used by people with a known illness and for trauma cases. One-fifth of calls fall into a category where the ambulance is not used often due to cancellation which is related to response time. Other factors such as the use of alternative transport are also important. Further study to identify factors that contribute to the non-transported category of calls is necessary if improvements in service quality are to be made.

## 1. Introduction

Barbados is a small island of 166 square miles (434 square km) with the highest point being approximately 1000 feet above sea level. The local population at July 2005 was estimated at 279,000 [1] with a population density of 1681 per square miles. It is the 15th most densely populated country in the world and is a popular tourist destination so that at any time, there are an estimated 300,000 people present. The island has a mixture of urban, suburban, and rural communities with 44% of the population living in the urbanised area [2]. 

The Barbados healthcare system is modelled from the UK healthcare system. Such that the Barbados Emergency Ambulance Service operated by the government is an emergency medical response service that is free to all Barbadians. The service operates a one-tier system active fleet of seven (7) vehicles manned by sixty (60) Emergency Medical Technicians and Paramedics. In Barbados, there are also a few nongovernmental ambulance services with a main focus on organised fee-for-service patient transport and mass event coverage. In addition The Barbados Defence Force has some ambulances that assist in times of extreme need, for example, during disasters. However, the majority of routine emergency calls on the island are responded by the Barbados Emergency Ambulance Service.

A major goal in Emergency Medical Services (EMSs) planning in Barbados as elsewhere [3] has been the need to minimize ambulance response times in the provision of definitive care. This is dependent on the efficient and targeted use of limited resources. The efficiency of services in other countries has been marred by the fact that the needs of a notable proportion of callers can be better met in ways other than by the dispatch of an emergency ambulance [4]. There is evidence that callers who live a greater distance from the ambulance station are somewhat compromised; these appear to be challenges common to the Barbados setting. Of some concern is the belief that in Barbados easy access to the ambulance has led to the abuse of the service which was intended for true emergencies as it is the case for the service offered in the United Kingdom (UK) [5, 6]. One study examined several social and practical points to see whether they would identify groups of patients who used the emergency service without medical need. They found that 38% of incidents were considered not to have medically warranted an ambulance call [6]. Comparison with Barbados was not possible as the routine data traditionally recorded in Barbados did not lend itself to easy collation for monitoring of these concerns, nor of evaluation of the service as a whole.

The main ambulance station (M) which functions as headquarters is located in the capital city Bridgetown. It previously serviced the needs of the entire island. An additional satellite station (S) operates from close to the center of the island at Arch Hall St. Thomas and services the northern and central portions of the country. These areas are more rural in nature and somewhat distant from the population centre. Establishment of this station is part of an ongoing effort aimed at addressing the need for improved response times to the more remote parts of the island with the intention of reducing waiting periods and client satisfaction. This was partially based on the perception that many calls ended in potential patients not being transported, although refusal would never be a provider led decision. With these objectives in view the ambulance service adopted a call taking system, the Medical Priority Dispatch System, from the United States of America (USA) [7] to help to more accurately dispatch services by the use of available routine information taken on calls to the centralized dispatch centre located at the central (Bridgetown) station. 

In this study we therefore sought to describe as a primary objective the reasons for non-transport of potential clients by analysing the current call type and the frequency of ambulance use in Barbados. The secondary objective is to identify any potential types of sources of misuse.

## 2. Materials and Methods 

### 2.1. Population

Calls arising from persons requesting service from the Barbados Emergency Ambulance Service.

### 2.2. Sample

All requests made by phone on behalf of persons seeking service from the Barbados Emergency Ambulance Service over the period from January to September 2005.

### 2.3. Procedures

All emergency calls to the Emergency Ambulance Service were received by dispatchers and recorded in the Action Register (Dispatch logbook). Data were collected by the use from a standardized international instrument (Medical Priority Dispatch System v11.2) [7]. This instrument was augmented by the addition of five local call types which were intended to capture the calls which were not covered by the Dispatch Protocol. The additional 5 types added were (1) Refused service (The patient decides not to utilise the services of the ambulance after its arrival at the scene.); (2) Not found (The ambulance crew is unable to locate the patient or incident.); (3) Left by other means (On arrival at the scene, it was found that the patient utilised some alternative means of transport.); (4) Cancelled (The patient or the caller decides not to utilise the services of the ambulance before its arrival at the scene.); (5) Not required (The ambulance arrives at a scene and a decision is taken by the attending team that it was not needed.) 

Recorded information included the date, time, nature and location of the calls and response time; however, response time was not categorized according to the acuity of complaint and a yearly average was calculated. Information was also gathered from the Patient Care Reports which were prepared by the responding Emergency Medical Technicians (EMTs) and Paramedics. These calls were then evaluated according to call location and placed into the 38 categories (referred to as types) as outlined in the Emergency Medical Dispatch Protocol [7] and the 5 local ones, by the Assistant Ambulance Officer on duty during the corresponding period. Data was collated and entered into a database. All data was derived from the Dispatch System only. The study was approved by the Institutional Review Board of the University of the West Indies/Ministry of Health Barbados. 

## 3. Statistical Analyses

The data are presented using descriptive statistics. Each type of call was assigned to one of seven categories (trauma, medical, environmental, pregnancy related, Psychiatric, miscellaneous, and non-transported). Correlations are examined using regression analyses. 

## 4. Results

Overall 8875 calls were made over the period from January to September 2005 to the ambulance service. Most calls were responded by the main station 86.9% compared to 13.1% at the satellite. When calls were ranked according to most frequently received at each station individually the rank order for the first ten types was roughly the same.  Table 1 shows each type of call as a percentage of the total calls responded sorted by the frequency of occurrence within categories. At the main station in the city of Bridgetown the highest percentage of calls came from the call type Sick person/known diagnosis followed by Breathing problems, Traffic accidents, and Cancelled calls. This pattern was generally repeated at the satellite station; however, the proportion of cases of pregnancy was higher and Heart problems and Haemorrhage stab/gunshot wound was lower. On average 7.5% of calls were from the call types Cancelled and 3.7 and 3.4% were from Not required and Refused (Table 1).

Over the period from January to September there appeared to be no seasonal variation to the number of calls; the mean ± (SD) per month was 986.1 ± (47.10) calls. Seventy-seven percent (77%) of calls were responded within 30 minutes, and most of these (75%) within 15 minutes. Average response time was the lowest in the central highly urbanized districts of the island.

### 4.1. Categories of Calls

When calls were placed in the seven categories, those categorised as medical (51.4%) comprised the highest proportion of all calls followed by Trauma (23.4%), Non-transported (19.0%), pregnancy (3.4%), psychiatric (1.1%), miscellaneous (1.0%), and environmental (0.7%). More Trauma calls were taken at the main station, Bridgetown, and a higher proportion of pregnancy related calls at the satellite, Arch Hall. Non-transported calls accounted for 19.0% of the calls by category (Table 1). 

### 4.2. Nontransported Calls

**Figure 1:**
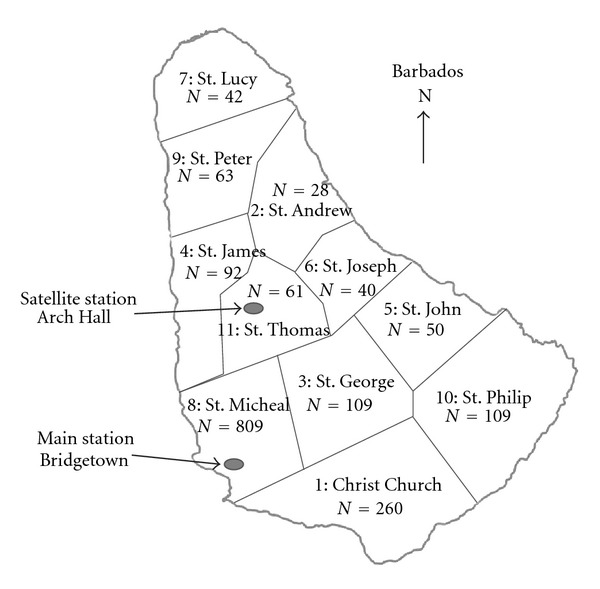
Schematic of the map of Barbados showing the number of Non-transported calls to the Barbados Ambulance Service by parish during January to September 2005.

Since the Non-transported category of calls was large we sought to examine the call types that contributed to response time and whether this was affected by geographical origin (parish) of the call. There was a significant overall difference in the frequency of the calls which contributed to this category. The call type Cancelled was the highest contributor to the Non-transported category of calls, this along with call type “Not required” accounted for 58% of the calls in the category (Table 1). Forty-eight (48)% of non-transported calls came from one central urban parish (Table 2); this represented a high proportion of non-transported calls by geographical area (20%).  Figure 1 shows the geographical location of the dispatch stations.

**Relationship of Nontransported Calls to Response Times**

**Figure 2:**
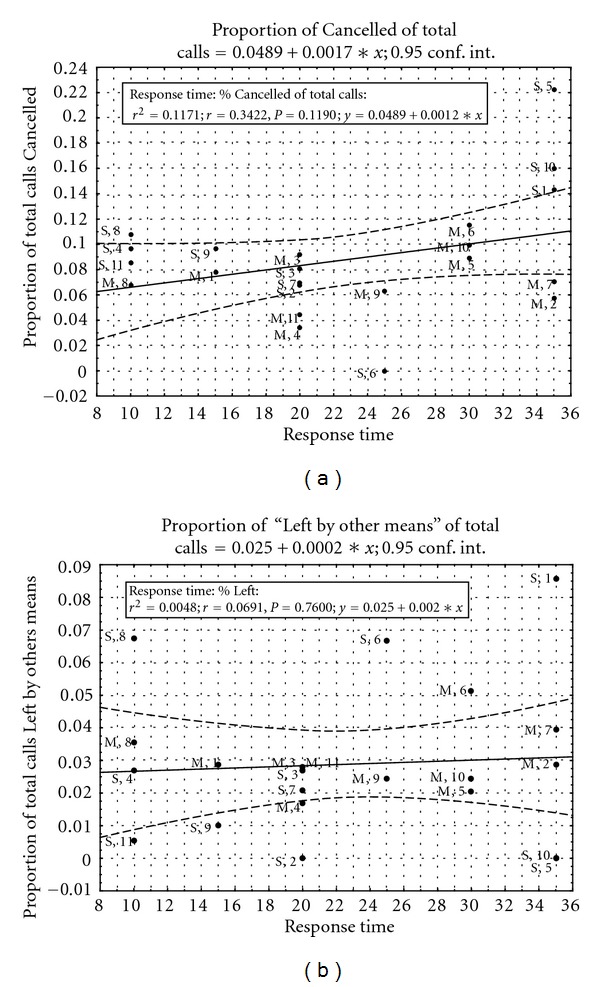
Correlation between response time and the call types Cancelled and Left by other means. Call types and average response time from the Barbados Ambulance Service from January to September 2005.

Correlations between the percentage of calls from all call types (*y*-axis) and the minimum response time recorded (*x*-axis) controlled for parish and station were calculated (Table 3 and Figure 2). Canceled service was significantly related to response time and Refused inversely so. Left by other means, Not found, and Not required were unrelated to response time.

## 5. Discussion

We are unaware of any other published studies on the emergency ambulance services of small island states. The geography presents peculiar challenges in that there is no access to assistance from neighbouring jurisdictions as would be the case for places with similar sized populations in other settings. Our data demonstrate as expected that a higher proportion of calls to the ambulance service are taken at the central (Bridgetown) station. In addition with a few exceptions types of calls are relatively equally apportioned to stations compared to the total number of calls taken by the station. Just over the half of the calls arise from subjects with a known diagnosis in the category medical which is similar to findings in the UK and elsewhere [8–10]. The types of unknown problem accounted for only 0.1% of calls overall and when call types were collapsed into categories the miscellaneous category accounted for around only 1% of all calls. This suggests that the instrument effectively captures the variety of conditions for which the ambulance service is called. 

The large proportion of calls in the category Non-transported demonstrated that the original instrument would have missed information on the service not related to patient injury or illness, accounting for 19% of calls. The most important contributors to this category were the call types Cancelled followed by Refused service and Left by other means. It is important to explore the reasons for such a high cancellation and refusal of service after having called for it, as any refusals are initiated by the potential patient or their caretaker and not provider-led. These and Left by other means may reflect calls that did not really need the use of the ambulance service. In the UK one study found that almost 16% of emergency ambulance calls were considered unanimously to be inappropriate [5]. Even in cases where the decision not to transport patients is taken by the emergency technician at the site of the incident there is evidence that most non-transported clients did not require immediate or urgent medical care [11]. Nevertheless this may also speak to what the client perceives as inadequate service, stemming from factors such as long waiting periods. 

Cancelled calls were the highest proportion of non-transported calls and this was directly related to response time, so that the longer a client had to wait the more likely they were to cancel the call. Notably, the category Left by other means was unrelated to response time. We postulate that there are two major factors contributing to a lack or relationship. Firstly these calls may be reclassified as Cancelled because this information is obtained after the ambulance had actually reached the scene, so that where response time is long it would give the callers more time to call and cancel. It may also reflect culture differences between rural and urban communities where rural peoples may be more likely to make a courtesy cancellation call. Secondly alternative vehicles are likely to be available in densely populated areas, where response time is short, diluting the relationship of Left by other means to response time. This is supported by the fact that despite a positive relationship of Cancelled calls and response time, 20% of Non-transported calls from the parish in which the main city is situated were Cancelled calls. We are unable to comment from our data on reasons why patients ultimately did not need the service. It is likely that the categories: Not found and Refused service, are more pronouncedurban phenomenon (calls for minor issues) as one might expect rural populations to be more robust with respect to minor injuries or incidents. 

Even though the Refused service category was inversely related to response time this is difficult to disentangle from the categories: Not found and Not required. 

A relatively high proportion of calls were responded within 10–15 minutes. Admittedly this tended to be in the most densely populated areas close to the ambulance stations. This is partially a product of strategic planning such that the placement of the stations close to the most densely populated areas permits better response to a larger number of calls. It must be borne in mind that the response times for acute situations were likely to be lower as the average response times included calls that were simply for transport, for example, the cases of diabetic foot that were nonemergency and were given low priority status. 

Alternatively, if the large non-transported category is a reflection of public dissatisfaction this may be arising from factors unrelated to the perceived deficiencies in the service, that are common to both stations. Prospective study needs to be conducted on distance of the station from the incident and on response time, which could not be answered in this study. 

Had we found a higher percentage of the non-transported calls to be in the Not found category we might have linked this to calls that were hoaxes. We therefore have little evidence from the ambulance data that the service although free is being misused by the public. However this can only be confirmed by the collection of information on patient outcomes after disposition at the accident and emergency department of the hospital. This is not the current practice but must be included in future routine data collection. 

The similarities between our service and that in other settings, suggest that many of the challenges to ambulance services are universal. We believe therefore that our local augmentation of the Dispatch instrument to include the Non-transported category has import not only for our service but obviously sheds light on an area of dispatch that is important but might not be used routinely. With testing and refinement of this portion of the instrument it can be useful to developing ambulance services over the world. 

It is likely that a proportion of the non-transported calls arise from long wait times experienced by clients. The number of ambulance units available is one unit per approximately 48,000 population, slightly better than the figure of one unit per 53,000 population that Braun et al. [12] found when examining 25 midsized cities with population between 400,000 and 900,000. So if there is a real waiting time problem, these figures indicate that one has to look for other contributing factors. These numbers can be lowered by improvements in the amount and quality of equipment and human resources provided. Also, improved access to training may provide for better practice methodologies. A good ambulance maintenance plan should also be in place. Qualitative study among crews may provided good starting points on where best to begin the process of change [13]. If we are to understand the effect of the prioritization of emergency ambulances on patient outcome the evidence must be collected. This problem echoes throughout the world [14]. 

### 5.1. Limitations

At the time of the study, response times are collated as averages for all call types so that it was not possible to distinguish between the response times dependent on priority status of the call. In addition no information was available on preferred routes especially when remote or heavily urbanized areas needed to be accessed. These factors would tend to make the ambulance service appear to operate less well than it actually does and would cloud a true picture of the strengths and weaknesses of the service in terms of ability to respond to public need. They can be addressed by the categorization of calls according to acuity and by the use of geographic information system (GIS) mapping to help to elucidate preferred routes. 

## Figures and Tables

**Table 1:** Distribution of calls by category, type, and station; the Barbados Emergency Ambulance Service (2005).

Call category	Call type	Calls satellite station	Calls main station	Total calls	% of category	% of total
Trauma	Traffic/transportation accidents	121	650	771	42.2%	8.7%
	Falls	33	280	313	17.1%	3.5%
	Traumatic injuries (specific)	25	203	228	12.5%	2.6%
	Haemorrhage/lacerations	12	209	221	12.1%	2.5%
	Assault/sexual assault	19	160	179	9.8%	2.0%
	Stab/gunshot/penetration trauma	3	81	84	4.6%	0.9%
	Animal bites/attacks	0	3	3	0.2%	0.0%
	Burns (scalds)/explosions	2	10	12	0.7%	0.1%
	Drowning (near)/diving/scuba accident	3	8	11	0.6%	0.1%
	Eye problems/injuries	2	2	4	0.2%	0.0%

	Total trauma	220	1606	1826	100.0%	20.6%

Medical	Sick person (specific diagnosis)	246	1510	1756	36.5%	19.8%
	Breathing problems	99	759	858	17.8%	9.7%
	Abdominal pain/problems	80	431	511	10.6%	5.8%
	Diabetic problems	39	321	360	7.5%	4.1%
	Chest pain (nontraumatic)	54	277	331	6.9%	3.7%
	Convulsions/seizures	42	279	321	6.7%	3.6%
	Unconscious/fainting (near)	26	225	251	13.7%	2.8%
	Stroke (CVA)	17	135	152	3.2%	1.7%
	Cardiac or respiratory arrest/death	12	109	121	2.5%	1.4%
	Heart problems/A.I.C.D	4	74	78	1.6%	0.9%
	Headache	9	64	73	1.5%	0.8%

	Total medical	628	4184	4812	100.0%	54.2%

Environmental	Allergies/envenomations (stings, bites)	4	52	56	87.5%	0.6%
	Carbon monoxide/inhalation/hazmat		5	5	7.8%	0.1%
	Electrocution/lightning	1	2	3	4.7%	0.0%

	Total environmental	5	59	64	100.0%	0.7%

Pregnancy	Pregnancy/childbirth/miscarriage	56	247	303	100.0%	3.4%

	Total pregnancy	56	247	303	100.0%	3.4%

Psychiatric	Overdose/poisoning (ingestion)	10	45	55	57.9%	0.6%
	Psychiatric/abnormal behaviour/suicide attempt	2	38	40	42.1%	0.5%

	Total psychiatric	12	83	95	100.0%	1.1%
Miscellaneous	Back Pain (nontraumatic)	6	76	82	89.1%	0.9%
	Unknown problem (man down)	2	3	5	5.4%	0.1%
	Choking	1	2	3	3.3%	0.0%
	Inaccessible incident/other entrapment (nonvehicle)		1	1	1.1%	0.0%
	Transfer/interfacility/palliative care		1	1	1.1%	0.0%

	Total miscellaneous	9	83	92	100.0%	1.0%
Nontransported	Cancelled	107	563	670	39.8%	7.5%
	Not required	48	282	330	19.6%	3.7%
	Refused service	39	263	302	17.9%	3.4%
	Left by other means	28	241	269	16.0%	3.0%
	Not found	7	105	112	6.7%	1.3%

	Total nontransported	229	1454	1683	100.0%	19.0%

Totals	All calls	**1159**	**7716**	**8875**		**100.0%**

Call types are not in the order in which they appear on the data intake instrument [7].

**Table 2:** Number of calls from each parish.

Parish*	Station	Total nontransported	Total calls	Response time (min)
1	m	249	1354	15
	s	11	35	35
2	m	12	105	35
	s	16	103	20
3	m	101	481	20
	s	8	37	20
4	m	39	351	20
	s	53	259	10
5	m	47	293	30
	s	3	9	35
6	m	36	156	30
	s	4	15	25
7	m	20	127	35
	s	22	143	20
8	m	763	3740	10
	s	46	148	10
9	m	30	205	25
	s	33	197	15
10	m	122	654	30
	s	7	25	35
11	m	35	250	20
	s	26	188	10

m: Main station; s: satellite station; *corresponds to number in Figure 2.

**Table 3:** The relationship of response time and the call types in the Non-transported category controlled for station and parish.

Call type	*r* ^2^	*r*	*P*	Regression line
Cancelled	0.1171	0.3422	0.1190	*y* = 0.0489 + 0.0017∗*x*
Left by other means	0.0048	0.0691	0.7600	*y* = 0.025 + 0.0002∗*x*
Not found	0.1864	−0.4317	0.0448	*y* = 0.0123 − 0.0003∗*x*
Not required	0.0854	0.2922	0.1869	*y* = 0.0265 + 0.0009∗*x*
Refused service	0.1144	−0.3382	0.1237	*y* = 0.0531 − 0.0011∗*x*
